# Ornamental Barberry Twigs as an Underexploited Source of Berberine-Rich Extracts—Preliminary Research

**DOI:** 10.3390/cimb46110787

**Published:** 2024-11-18

**Authors:** Michał Miłek, Małgorzata Dżugan, Natalia Pieńkowska, Sabina Galiniak, Mateusz Mołoń, Wojciech Litwińczuk

**Affiliations:** 1 Department of Chemistry and Food Toxicology, Institute of Food Technology and Nutrition, University of Rzeszow, Ćwiklińskiej 1a, 35-601 Rzeszow, Poland; mmilek@ur.edu.pl; 2 Institute of Medical Sciences, University of Rzeszow, Warzywna 1a, 35-310 Rzeszow, Poland; npienkowska@ur.edu.pl (N.P.); sgaliniak@ur.edu.pl (S.G.); 3 Institute of Biology, University of Rzeszow, Zelwerowicza 4, 35-601 Rzeszow, Poland; mmolon@ur.edu.pl; 4 Department of Physiology and Plant Biotechnology, Institute of Agricultural Sciences, Environment Management and Protection, University of Rzeszow, Ćwiklińskiej 2, 35-601 Rzeszow, Poland; wlitwinczuk@ur.edu.pl

**Keywords:** berberine, ornamental barberry, twigs, polyphenols, HPTLC, anticancer

## Abstract

Berberine is a natural substance obtained from the roots of common barberry which, due to its strong pharmacological activity, is a commonly tested ingredient of dietary supplements. However, ornamental barberries, which are widely available, have not been considered as a source of berberine so far. The research aimed to check whether the ornamental barberry leaves and twigs could be used as an easily accessible raw material for obtaining natural berberine-rich extract with biological activity. Twigs and leaves of seven cultivars of ornamental barberry extracts were assessed for their polyphenol content, antioxidant potential (FRAP and DPPH), and berberine content using high-performance thin layer chromatography (HPTLC). As a reference, commercially available roots of *Berberis vulgaris* were used. For the next step, selected extracts (two with high and two with low berberine content) were tested on three cell lines (HaCaT, A375, Caco-2) using neutral red assay, and pure berberine sulfate (1–100 μg mL^−1^) was used as a control. Although the antioxidant potential of aqueous–methanol extracts of tested barberry was higher for the leaves than for the twigs, the berberine content was determined only in the twig extracts (from 42 to 676 mg 100 g^−1^). Studies on cell lines have shown the general toxicity of barberry extracts, but the observed effect was not directly correlated with the content of the alkaloid. However, the extract showed greater activity compared to an analogous dose of pure berberine, suggesting a significant effect of the matrix composition. For the first time, it was shown that the twigs of selected cultivars of ornamental barberry can be considered as a promising berberine source for the pharmaceutical industry to develop new effective formulations. However, these findings require further studies.

## 1. Introduction

A large genus of barberry (*Berberis* sp.) includes approximately 400–450 species of deciduous and evergreen shrubs found in Europe, America, and Africa [1,2]. Only one species—European (common) barberry (*B. vulgaris* L.) grows wild in Poland. It is also common in Europe, North Africa, the Middle East, and Central Asia [2]. However, it is of no great importance in horticulture (and as an alternative crop) as it is susceptible to fungal diseases, including rust and powdery mildew. What is worse, it is the primary host of *Puccinia graminis* Pers. which causes a cereal disease called stalk rust of cereals and grasses [3,4]. Other species, among others Thunberg’s barberry (*B. thunbergii* DC.), are not as susceptible to diseases and offered as ornamental plants in hundreds of cultivars with varied shapes, growth strength, and colorful leaves (different shades and combinations of yellow, green, and red). It is well known that the color of leaves is related to the presence of various polyphenols and carotenoids. Flavonoids and phenolic acids are present in leaves and flowers, while fruits are abundant in anthocyanins [5]. More than 20 alkaloids (including berberine, palmatine, oxyberberine, isocoridine, lambertine, berbamine, jatrrorhizine) were found in the barberry plant [2,5]. Chemically, they belong to the groups of protoberberine, isoquinoline, and bisbenzylisoquinoline alkaloids [5]. Both groups of secondary metabolites have various properties beneficial for medicinal uses. The main areas of ethnomedical applications were the territories of present-day China, India, Turkey, and Iran [6]. The traditionally used effects of barberry preparations include cholagogue, stomachic, laxative, diaphoretic, antipyretic, and antiseptic. They were also used for skin problems, hemorrhoids, and varicose veins [1,5]. Modern pharmacological studies confirm the antidiabetic, anti-inflammatory, wound-healing, anti-allergic, immunomodulatory, hepatoprotective, and anticancer effects of barberry. It also has strong antioxidant and antimicrobial effects [5,7]. Barberry also plays a role in the food industry as a source of fruit used to produce drinks, liqueurs, sauces, desserts, and jellies [6].

**Figure 1:**
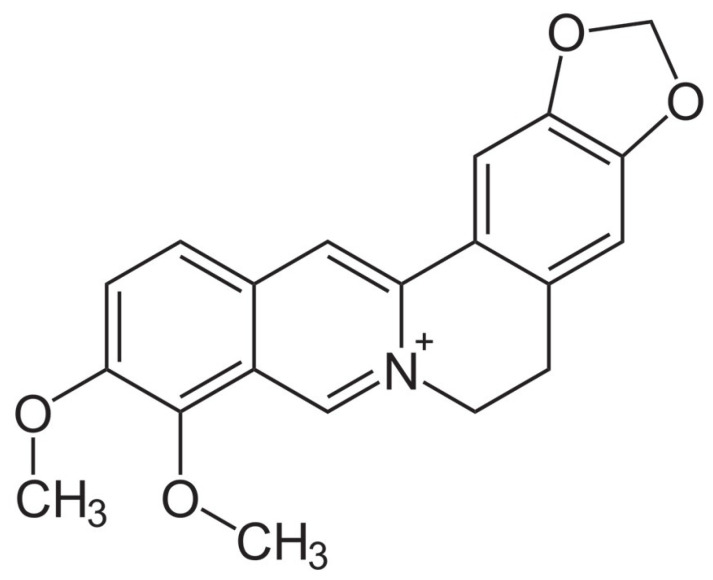
Berberine structure.

The main bioactive substance of barberry, named after this plant, is berberine (Figure 1). It is classified as an isoquinoline alkaloid. In addition to the *Berberis* genus, it is also present in other plants from the Annonaceae, Papaveraceae, Menispermaceae, Ranunculaceae, and Rutaceae families [8]. The other genera and species of plants containing this alkaloid include, among others, *Annickia* sp., *Jeffersonia diphylla*, *Mahonia* sp., *Tinospora sinensis*, *Agremone* sp., *Chelidonium majus*, *Corydalis* sp., *Papaver* sp., *Coptis* sp., *Hydrastis canadensis*, and *Phyllodendron* sp. [8]. Berberine can be extracted from plant material using various techniques, most commonly using methanol, ethanol, chloroform, and other solvent mixtures [8]. Pure berberine can be obtained from extracts by lowering the pH through the addition of concentrated acid (hydrochloric or sulfuric) and cooling to cause crystallization [9]. In dietary supplements, it is most often used in the salt form as chloride or sulfate, with better solubility in water [10]. Moreover, due to extremely low berberine bioavailability, various nano-carriers and enhancers are applied [10,11]. Various analytical techniques have been used to determine berberine in plant material or drug preparation (HPLC, TLC, Capillary electrophoresis, GC-MS, colorimetry), among them TLC is distinguished by low cost, ease of implementation, good accuracy and stability [10].

In addition to berberine and other alkaloids, polyphenols constitute an important group of barberry phytochemical components: flavonoids (luteolin, isorhamnetin and quercetin derivatives) as well as anthocyanins (peonidin, cyanidin, petunidin, malvidin, leonidin and their derivatives) [1]. Barberry fruits also contain carotenoids [1]. The HPTLC technique, a more advanced version of thin layer chromatography, is increasingly used to analyze phytochemical profiles of plant extracts, as it allows for direct comparison of up to 20 samples on one chromatographic plate [10,12,13].

Berberine, in addition to its antioxidant and antimicrobial effects, also exhibits immunomodulatory, cardioprotective, hepatoprotective, and renoprotective effects [8,14,15]. It is used in cardiovascular and digestive diseases, diabetes, and skin problems. Several possible mechanisms for lowering blood glucose levels have been identified [8]. Also, several mechanisms of the anticancer action of this alkaloid have been identified, including inducing cell cycle arrest, inhibition of telomerase, induction of apoptosis, induction of anti-inflammatory cytokines, inhibition of angiogenesis, and ROS-mediated mechanisms [8,16]. Great hopes are attached to its use in the treatment of neurodegenerative diseases [17,18]. Due to this, new sources of berberine are being sought, and attempts are being made to synthesize it and its analogs with potential pharmacological activity [19,20,21,22,23]. The bioactivity of berberine is assessed primarily in vitro on cell lines, but many clinical trials have already been conducted [24]. According to market reports, the global berberine market, mainly for dietary supplements, pharmaceutical and cosmetic industry, was estimated at USD 852.3 million in 2023 and is expected to grow to USD 1845.4 million in 2033, with a CAGR of 8.9% during the forecast period from 2024 to 2033 [25]. Taking into account such data, the search for new sources of this bioactive substance is economically justified.

The wealth of bioactive compounds is contained not only in easily accessible fruits but also in the leaves, twigs, and roots of barberries. More and more often, the acquisition of these raw materials is abandoned due to the significant reduction in the population of wild barberry [26]. The question arises whether the leafy twigs of ornamental barberry can be considered as good source of berberine as the roots of *B. vulgaris*. Thus, the work aimed to select ornamental cultivars of barberry that would be a more efficient and cheaper source of berberine, and at the same time, its cultivation would be safer for crops than common barberry. To our knowledge, this is the first time that twigs and leaves of selected ornamental barberry varieties have been studied. Initial studies have focused on assessing the bioactivity of full extracts from the tested plant materials.

## 2. Materials and Methods

### 2.1. Plant Material

The plant samples (several-month-old twigs, of 10–50 cm length and max 8 mm diameter) were manually collected from the central part of three healthy shrubs of each clone at the end of September (before mass fruit ripening). The 7-year barberry (*Berberis* sp.) shrubs were growing in the light shade in the private collection of Wiesław Więcek Nursery of Ornamental Trees and Shrubs (Stobierna 38, 36-002 Jasionka, Poland; 50.143, 22.072). Most of the clones belonged to the *Berberis thunbergii*, two to the *Berberis koreana*, and one was an interspecific (*B. thunbergii* × *B. vulgaris*) hybrid (Table 1). They differed in shrub growth intensity and shape, and mainly in the color of the leaves, from yellow through green, red to purple. The color of the leaves may have changed during the growing season.

The collected leafy twigs were dried for 10 days in the open air at room temperature. Then, twigs and leaves were separated. The dry plant materials were stored in a dry place closed in paper bags protected from light until the analyzes (up to six months) in a controlled humidity (25–30%) and temperature (20–22 °C) chamber. Two samples of *Berberis vulgaris* root bark (conventional and organic) were purchased from Nat Vita (Długołęka, Poland) company as a control sample of a herbal source of berberine. Berberine sulfate (99% purity) was purchased from Aliness (Ostrówiec, Poland).

### 2.2. Extracts Preparation

Extracts were prepared according to a modified procedure of Alam et al. [27]. Briefly, 5 g of ground, dried plant material was flooded with 25 mL of 70% ethanol. Extraction was carried out in an ultrasonic bath (700 W; Sonic-10, Polsonic, Warsaw, Poland) twice for 20 min at a temperature of about 50 °C. Then, the extracts were filtered through paper and subjected to chemical analyses. For biological studies, they were concentrated to about 1/3 volume, removing ethanol in a vacuum rotary evaporator (RVC 2–18 CDPlus, Martin Christ, Osterode am Harz, Germany) and lyophilized (Alpha 1–2 LD plus, Martin Christ, Osterode am Harz, Germany) for 48 h to obtain a dry extract.

### 2.3. Total Phenolic Content

Total phenolic content in crude extracts was measured according to Dżugan et al. [27] using the Folin–Ciocalteu method. The results were expressed in mg of gallic acid equivalents per g of dry weight of raw material.

### 2.4. Antioxidant Potential

The antioxidant potential of crude extracts was assessed using FRAP and DPPH methods according to Dżugan et al. [28]. The results were expressed in μmol of Trolox equivalents per g of dry weight of raw material.

### 2.5. HPTLC Determination of Berberine

The berberine content in the extracts was assessed by HPTLC according to the modified procedure of Alam et al. [26]. Extracts in a volume of 2 µL were applied to HPTLC Silica Gel 60 F254 plates (20 cm × 10 cm) (Merck, Darmstadt, Germany) using a Linomat 5 automatic applicator (Camag, Muttenz, Switzerland). The chromatogram was developed using a mobile phase composed of n-propanol, formic acid, and water (90:1:9, *v*/*v*/*v*) in the automatic developing chamber (ADC2, Camag). After development, the plate was visualized under 366 nm UV light using a TLC Visualizer (Camag). Quantitative analysis was performed based on the standard curve for berberine sulfate (250 μg mL^−1^ in methanol) applied in a volume of 2 to 8 µL (0.5 to 2 μg of standard). A standard curve (y = $\frac{2.812\times 10^{-2}x}{5.958\;10^{-7}+x}$, R^2^ = 0.9906) was constructed based on the peak area of the chromatograms generated by the software (Vision CATS, Camag). The analyses were conducted under constant conditions of temperature (22 °C) and relative humidity (33%). For the method, repeatability and accuracy were established by applying three selected standard concentrations on the same day (3 repetitions) and on 3 different days; both parameters did not exceed 0.3% RSD. Based on the signal-to-noise ratio (S/N), the limit of detection (LOD, S/N = 3) was established as 2.5 μg mL^−1^ and the limit of quantitation (LOQ, S/N = 10) as 12.5 μg mL^−1^.

### 2.6. Cell Culture

Cytotoxicity was studied in three human cell lines—immortalized keratinocytes HaCaT, human malignant melanoma A375, and human colorectal adenocarcinoma cells Caco-2. All used cell lines were obtained from ATCC (American Type Culture Collection, Manassas, VA, USA). Cells were cultured in DMEM/F-12 with 10% heat-inactivated FBS (HaCaT and A-375) or 20% heat-inactivated FBS (Caco-2), 1% *v*/*v* penicillin-streptomycin and incubated at 37 °C under conditions of 5% CO_2_, 95% humidity (Binder, Germany). The growth medium was changed twice a week, cells were passed at 80–90% confluence using 0.25% trypsin 0.03% EDTA in calcium and magnesium-free PBS.

### 2.7. Cytotoxicity Assay

Cells were seeded in a 96-well clear plate at a density of 7.5 × 10^3^ cells/well (HaCaT) and 1 × 10^4^ (A-375 and Caco-2) in 100 µL culture medium and allowed to attach for 24 h at 37 °C. The cells were treated with tested sterile extracts or berberine for 24 h. Non-treated cells were used as a control. After exposure, the cytotoxicity test—neutral red assay (NR)—was performed according to Repetto et al. [29]. Briefly, the medium with the compound tested was removed and replaced with a 2% neutral red solution in cell culture medium (100 µL/well), the plate was incubated at 37 °C for 1 h. The cells were then rinsed with warm PBS and a permeabilized solution (50% distilled water, 49% ethanol 96%, and 1% glacial acetic acid) (100 µL/well).

The plate was shaken (800 rpm) at room temperature for 25 min (Heidolph Inkubator 1000, Germany). Absorbance was measured at 540 nm against 620 nm using the TECAN Infinite 200 microplate reader (Tecan, Grödig, Austria). The results were presented as a percentage of control counted from 9 replicates.

### 2.8. Statistical Analysis

The results were presented as mean values ± SD (total phenolic content, antioxidant potential and berberine content) or interquartile range (IQR; 25%–75%) (cytotxicity assay). To evaluate the significance of differences between compared samples, one-way ANOVA and Tukey’s test were performed after prior confirmation of the normality of the data distribution. To evaluate the differences between the control and the treated cells, one-way ANOVA and Dunnett’s post hoc tests were utilized. A *p*-value of less than 0.05 was deemed to indicate statistical significance (for the confidence interval level of 95%). The statistical evaluations were performed using the Statistica 13.3 (Statsoft, Tulsa, OK, USA) software.

## 3. Results and Discussion

### 3.1. Total Phenolic Content and Antioxidant Potential of Extracts

The total phenolic content and antioxidant potential of the tested barberry twigs are summarized in Table 2.

Higher values of polyphenol content and correlated (Pearson’s coefficients above 0.9) antioxidant capacity were found for barberry leaves (Table 3). The polyphenol content ranged from 49.25 mg GAE g^−1^ dry weight for *B. thunbergii* ‘Powwow’ to 136.64 for the ‘Golden Ring’ cultivars. This may be related to the leaf color; the ‘Golden Ring’ cultivar is characterized by dark red leaves, so the TPC index may also include anthocyanins present in them. In the case of twigs, the polyphenol content was lower but more even, between 15 and 32 mg GAE g^−1^ of raw material. Since barberry leaves and twigs, especially ornamental varieties, are not typical herbal raw materials, it is difficult to find data on the content of phenolic compounds and antioxidant potential. For *B. vulgaris*, the polyphenol content in leaves was reported at 58.5 mg GAE g^−1^, similar to the stems—57.7 mg g^−1^ [14]. The bark of barberry roots examined for comparative purposes was characterized by a much lower content of phenolic compounds and antioxidant potential that was several times lower than that of other plant raw materials. This suggests that above-ground parts of these plants may be valuable sources of antioxidants. Previously, the roots, twigs, and leaves of two species of barberry growing in different locations in Croatia were compared. The content of phenolic compounds was of a similar order of magnitude as in our case; furthermore, the IC50 value for the DPPH method increased in the order: of root, twigs, leaves, which means that leaves have the highest antiradical potential [30]. This trend was observed for both *B. vulgaris* and *B. croatica*. This confirms the observations described above, regardless of the species and cultivar of the plant. Another study, for *B. vulgaris*, however, indicated that the root extract was a more potent free radical scavenger (44.3% inhibition) than the leaf extract (21.4%) [31]. A similar trend was described for other methods: ABTS scavenging and β-carotene bleaching. Some studies also indicate the antioxidant potential of berberine in vitro [9,32], although our studies did not confirm this.

### 3.2. Berberine Content

**Figure 2:**
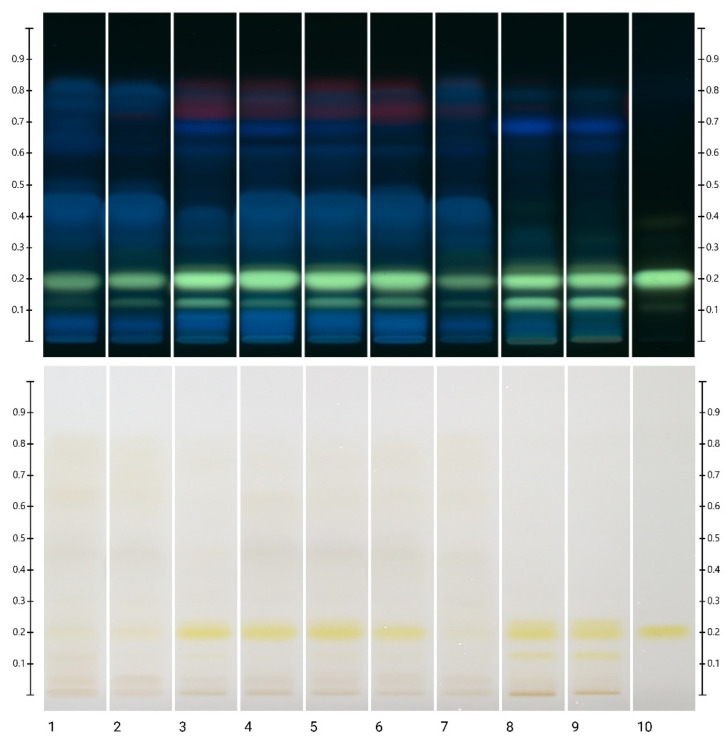
HPTLC analysis of berberine content in twig extracts. Top visible in 366 nm UV light, bottom in visible light. Tracks: 1—‘NN’, 2—‘Superba’, 3—‘Powwow’, 4—‘Golden Carpet’, 5—‘Red Pillar’, 6—‘Golden Ring’, 7—‘Red Tears’, 8—root bark, 9—*Berberis vulgaris* (organic) root bark, 10—berberine standard.

Berberine is usually determined by HPLC or UHPLC, but an attempt is being made to simplify the analysis by introducing the HPTLC method [13,33,34,35]. Figure 2 shows the results of the separation of ornamental barberry twig extracts and *B. vulgaris* root bark extract by the HPTLC method. The yellow band at Rf = 0.20 in UV 366 nm corresponds to berberine. This alkaloid was not detected in leaf extracts. For all extracts, an additional band at Rf = 0.12 was visible, which may be attributed to palmatine [35]. It is the second most abundant alkaloid determined in barberry [36]. In the case of samples of ‘Powwow’, ‘Golden Carpet’, ‘Red Pilar’, ‘Golden Ring’, and common barberry roots, bands from another alkaloid (Rf = 0.22) were visible above the berberine band.

**Figure 3:**
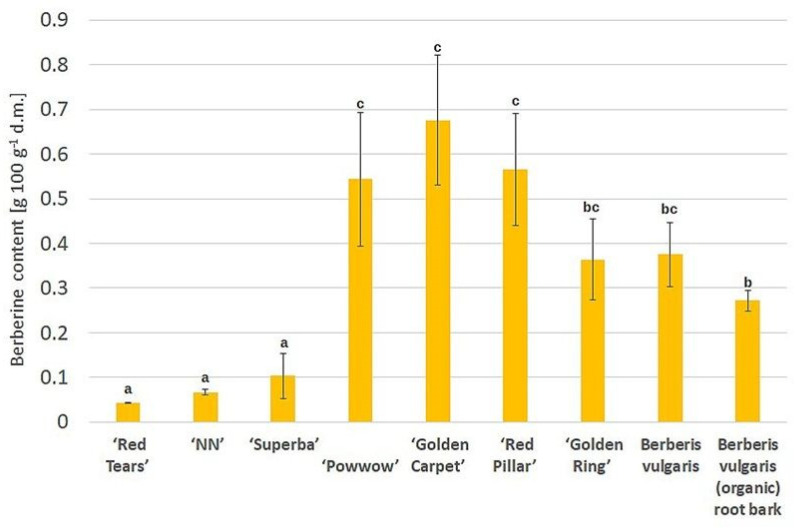
Berberine content in twigs of tested barberry cultivars compared to reference material (*B. vulgaris* root bark). The bars represent standard deviation; a,b,c—means marked with different letters are significantly different (*p* < 0.05).

Based on the fluorescence intensity of berberine bands in UV 366 nm, a standard curve was constructed and the alkaloid content in the extracts tested was calculated. The results for twigs of tested ornamental cultivars and *B. vulgaris* root bark are presented in Figure 3.

The lowest values for *B. koreana species* (0.042–0.067%) compared to *B. thunbergii* varieties (0.364–0.676%) were obtained. The intermediate value for the ‘Superba’ hybrid (0.103%) was also found. Compared to tested *B. vulgaris* root bark, the content found in all *B. thunbergii* cultivars was on average 66% higher. Berberine content has previously been studied mainly in the root bark, less frequently in the stem bark of various barberry species. A similar value, determined using HPLC-DAD-MS, for the roots of *B. vulgaris* was given by Vilinsky et al. [37] (0.426%); for *B. thunbergii* of an unspecified cultivar, these authors offered an even higher value (1.377%). For *B. lycium* roots, values in a similar range (0.203–1.134%) were reported by Chaudhary et al. [35] and even higher for *B. lycium* and *B. aristata* (2.6–3%) by Andola et al. [13]. For *B. aristata* stem bark, depending on the geographical origin of the plant, Ahamad et al. [34] reported a berberine content of as much as 6.14–9.44%. In turn, the berberine content (LC-MS/MS quantitation) in cortex from 0.058 mg g^−1^ (*B. gagnepainii*) to 1.15 mg g^−1^ (*B. pruinosa*) was reported by Tuzimski et al. [36]. In some cases, quantification of berberine in extracts was also carried out by the HPTLC technique [13,34,35]. The reported higher berberine contents in the roots and bark of barberry species other than *B. vulgaris* may suggest their potential use as a source of berberine, as well as the use of other parts of the plant.

### 3.3. Cytotoxic Effect of Ornamental Barberry Extracts

Discovering effective natural compounds with potent anticancer activity is a crucial area of research. Natural products have long been a rich source of novel chemotherapeutic agents, with many approved drugs and drug candidates originating from plant, marine, or microbial sources [38,39]. Cytotoxicity assays are a fundamental tool in this process, allowing researchers to assess the ability of natural extracts or purified compounds to selectively kill cancer cells [40,41]. One of the key advantages of natural products as a source of anticancer drugs is their diverse chemical structures and modes of action [42]. Many natural compounds exhibit cytotoxicity through different mechanisms, inducing oxidative stress, or modulating key signaling pathways in cancer cells [39]. Carefully designed cytotoxicity assays can help elucidate these mechanisms and guide the development of natural product-derived therapeutics.

Taking the above into account, selected extracts from ornamental barberry twigs were subjected to cytotoxicity studies using three cell lines. Two extracts with high (‘Golden Carpet’ and ‘Golden Ring’) and two with low (‘Superba’ and ‘Red Tears’) berberine content were tested with the use of three human cell lines—immortalized keratinocytes HaCaT, human malignant melanoma A375 and human colorectal adenocarcinoma cells Caco-2. In addition, based on cultivar differences an attempt was made to determine the mechanism of the observed effect and to identify key components of extracts.

**Figure 4:**
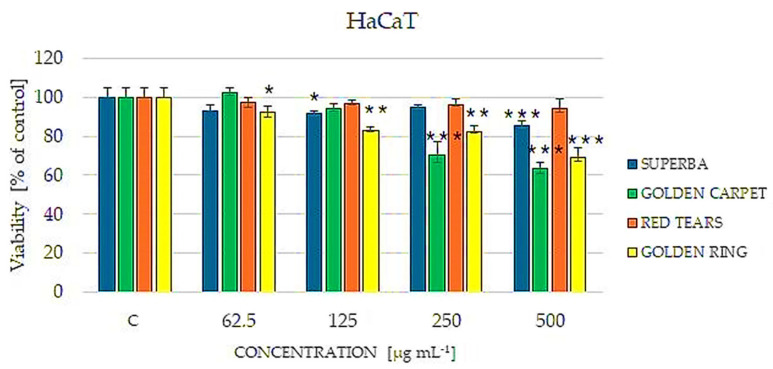
Cultivar-dependent effect of selected barberry extracts (‘Superba’, ‘Golden carpet’, ‘Red Tears’, ‘Golden ring’) on the viability of human keratinocytes HaCaT cells estimated by the Neutral Red assay. The cells were treated with extracts in concentrations of 62.5, 125, 250 and 500 μg mL^−1^. Non-treated cells were used as a control (C). Data are expressed as median from at least three independent experiments. Error bars represent 25% and 75% percentiles. Statistical significance was assessed using one-way ANOVA and Dunnett’s post hoc test (* *p* < 0.05, ** *p* < 0.01, *** *p* < 0.001).

As shown in Figure 4, only the ‘Red Tears’ extract did not have a statistically significant effect on HaCaT keratinocytes at concentrations up to 500 µg mL^−1^. In contrast, the other three extracts analyzed, namely ‘Superba’, ‘Golden Carpet’, and ‘Golden Ring’, exhibited dose-dependent toxicity towards the treated keratinocyte cells. Interestingly, the absence of berberine in ‘Red Tears’ correlates with its ineffectiveness, suggesting that berberine is a key factor significantly limiting the viability of keratinocyte cells. Conversely, the high berberine content in ‘Golden Carpet’ and ‘Golden Ring’ and the associated negative effects at higher doses (*p* < 0.001) indicate the potential cytotoxic properties of this alkaloid at elevated concentrations. In addition to berberine, the extracts also contain numerous polyphenolic compounds, which can also affect the bioactivity of the extracts in vitro. The ‘Red Tears’ cultivar was characterized by a significantly lower TPC value than the others tested on the HaCaT line. The weaker effect of the extract of this variety, even at a high concentration of the extract, may result from a lower content of polyphenols or matrix effects, weaker synergy of this class of metabolites with berberine.

**Figure 5:**
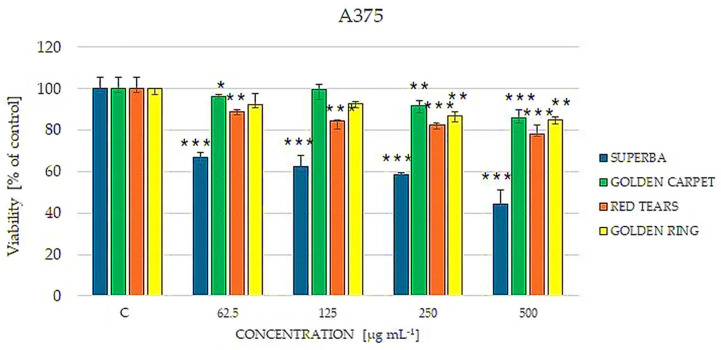
Cultivar-dependent effect of selected barberry extracts (‘Superba’, ‘Golden carpet’, ‘Red Tears’, ‘Golden ring’) on the viability of human malignant melanoma A375 cells estimated by the Neutral Red assay. The cells were treated with extracts in concentrations of 62.5, 125, 250, and 500 μg mL^−1^. Non-treated cells were used as a control (C). Data are expressed as median from at least three independent experiments. Error bars represent 25% and 75% percentiles. Statistical significance was assessed using one-way ANOVA and Dunnett’s post hoc test (* *p* < 0.05, ** *p* < 0.01, *** *p* < 0.001).

Subsequently, the impact of the analyzed extracts on the viability of A375 melanoma cancer cells was investigated. As shown in Figure 5, the ‘Superba’ extract exhibited the strongest cytotoxic effect among the analyzed extracts. Even the lowest concentration of the ‘Superba’ extract had a statistically significant impact on the cytotoxicity of A375 cells (*p* < 0.001). At a concentration of 500 µg mL^−1^, cell viability decreased to 44%. Interestingly, compared to HaCaT cells, A375 cells were sensitive to the ‘Red Tears’ extract, with viability dropping to 80% at concentrations of 250 and 500 µg mL^−1^ (*p* < 0.001). The extracts of ‘Golden Carpet’ and ‘Golden Ring’ abundant in berberine had the least impact on A375 cells, showing dose-dependent effects; at the highest concentrations analyzed, the cytotoxic impact on viability was statistically significant. Therefore, our preliminary research suggests that the use of barberry extracts in the treatment of melanoma may be reasonable, however, the observed cytotoxic effect does not seem to be directly related to the berberine content. However, a previous study reported the inhibitory effect of pure berberine on the migration of melanoma cancer cells [43].

**Figure 6:**
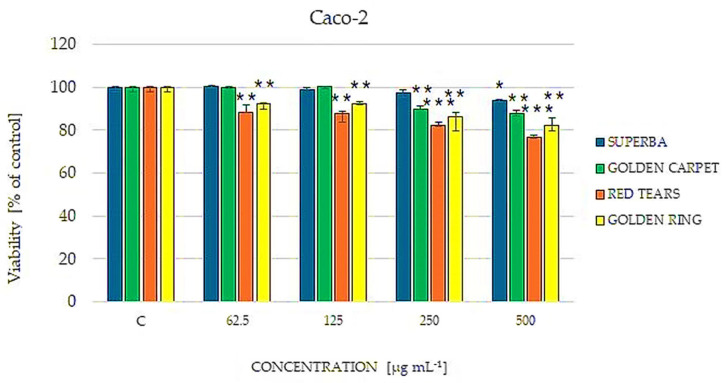
Cultivar-dependent effect of selected barberry extracts (‘Superba’, ‘Golden carpet’, ‘Red Tears’, ‘Golden ring’) on the viability of human colorectal adenocarcinoma cells Caco-2 estimated by the Neutral Red assay. The cells were treated with extracts in concentrations of 62.5, 125, 250 and 500 μg mL^−1^ Non-treated cells were used as a control (C). Data are expressed as median from at least three independent experiments. Error bars represent 25% and 75% percentiles. Statistical significance was assessed using one-way ANOVA and Dunnett’s post hoc test (* *p* < 0.05, ** *p* < 0.01, *** *p* < 0.001).

As Caco-2 colorectal cancer cells have been more frequently used in the examination of the mechanism of berberine activity, we also investigated the impact of the analyzed extracts on Caco-2 cells. As shown in Figure 6, the utilized concentration of 500 μg mL^−1^ had a toxic effect on these cells in the case of all analyzed extracts. A particularly significant impact was noted for berberine-rich ‘Golden Carpet’ and ‘Golden Ring’ extracts but also for ‘Red Tears’ extract. In conclusion, Caco-2 cells were sensitive to barberry twig extracts, but it has not been confirmed that it is dependent on the berberine content.

To confirm the tendency observed for barberry extracts cytotoxicity we have undertaken similar studies for pure berberine in the concentration range 0.001–0.25 mg mL^−1^ with the use of previously analyzed human cells. Taking into account the concentration of berberine in the extracts used (from 1 to 16 µg mL^−1^) Table 3 shows the effect for comparable doses of pure berberine sulfate. The dose of 100 µg mL^−1^ unequivocally exhibited cytotoxic activity against keratinocytes and both cancer cells used in the study. Interestingly, this cytotoxic effect was significant in the case of the HaCaT and A375 cell lines for 10-fold lower dose.

No direct dependence of the polyphenols content in the extracts on the in vitro effects has been observed, but they are important components of the extract matrix and may shape the bioactivity of the entire extract through synergistic or antagonistic interactions.

Subsequently, calculations were performed to compare cytotoxicity effects taking into account the berberine content in the highest dose of extracts (500 mg mL^−1^) (Table 4). The comparison carried out showed that tested extracts in general exhibited stronger cytotoxicity than comparable doses of pure berberine. Thus, it could be concluded that berberine in the composition of other phytochemicals that occurred in barberry extracts has a different cytotoxic effect compared to pure berberine. In this respect, the results obtained are promising and require continuation with higher doses of the extract in cytotoxicity assays on cell lines. It should be mentioned, however, that berberine and its derivatives can activate or enhance lysosomal activity in cells [44], hence the applied Neutral Red test may have certain limitations and the results should be confirmed using other procedures.

Recent studies have highlighted the potential of berberine as a promising anticancer agent, with the ability to inhibit the proliferation and induce the death of cancer cells [45]. Berberine has been shown to exert antiproliferative effects on a variety of cancer cell types, including hepatoma, colon, epithelial ovarian, and breast cancer cells [46,47]. In addition to its antiproliferative effects, berberine has also demonstrated proapoptotic properties [48,49,50]. Moreover, it has been reported to modulate the production of inflammatory mediators, such as tumor necrosis factor-α, in cancer cells [50]. In addition to its direct antiproliferative effects, berberine has also been reported to exhibit other beneficial properties that may contribute to its anticancer potential, like modulating epigenetic mechanisms [45].

However, many studies on the pharmacological potential of berberine have been performed for pure compound and do not take into account the influence of additional components contained in the barberry extract. Meanwhile, the positive matrix effect on phytochemical bioaccessibility can be considered as was shown in the case of organosulfur components of garlic [51]. This is especially important due to the low solubility and the low absorption of berberine itself which represent a limiting factor to its activity. The majority of efforts were addressed to improve those liabilities to reduce the high dosages that result in gastrointestinal adverse events [52,53]. The absorption of berberine from extracts of ornamental barberry twigs may be better due to enhancing the impact of the matrix, but this requires further research using in vitro digestion, analogously to the study of Petrangolini et al. [54].

There are known examples of extracts having a stronger effect than individual metabolites. In terms of antimicrobial properties, it has been previously found that the full plant extract containing berberine was more active than the isolated alkaloid [55]. Similarly, a stronger effect of crude barberry extract than berberine chloride was demonstrated in the α-glucosidase inhibition test, whereas the effect on acetylcholinesterase was the same [56]. Some research suggests that barberry extracts can have beneficial effects on the cardiovascular system. They may help in lowering blood pressure, reducing cholesterol levels, and improving overall heart health [57]. One of the most frequently studied biological effects, including clinical studies, is the antidiabetic effect of barberry extracts and berberine itself. Promising results have been obtained for berberine as an active substance, and the proposed mechanisms of action include increasing insulin sensitivity, modulating gut microbiota, activating the adenosine AMPK pathway, promoting intestinal glucagon-like protein-1 secretion, stimulating glycolysis, inhibiting gluconeogenesis in liver, and upregulating hepatic low-density lipoprotein receptor mRNA expression [58]. Moreover, extracts of *Berberis* sp. and other plants containing these alkaloids have also been studied in this field. However, the effects of the studies were not as directly promising as for pure berberine [58]. Numerous reports confirm the usefulness of both berberine and plant extracts rich in it in the treatment of the metabolic syndrome [58]. As confirmation of the positive effect of barberry extracts in the treatment of cancer in the studies by Rigillo et al., it was demonstrated that *B. aristata* extracts containing berberine and other protoberberine-type alkaloids inhibited the migration of cancer cells without any effect on healthy cells [59].

Overall, the available evidence suggests that ornamental barberry twig extract is a promising natural source of active berberine. Moreover, the full extract of twigs of selected ornamental barberry varieties seems to have greater activity compared to the analogous dose of the reference berberine sulfate. This may indicate the occurrence of a beneficial effect of the components of the complex matrix of the extract. The next step should be the confirmation of the bioactivity of pure berberine isolated from the tested raw materials. Well-evidenced ability of berberine to inhibit the proliferation of various cancer cell types, as well as its potential to modulate key signaling pathways and epigenetic mechanisms, make the studied raw material an intriguing target for further investigation and potential clinical trial. Moreover, due to the widespread and greater safety of ornamental barberry cultivation compared to *B. vulgaris*, the obtained results are promising and can be implemented.

## 4. Conclusions

Using quantitative thin-layer chromatography, it was shown for the first time that ornamental barberry twigs collected before fruit ripening contain berberine (from 0.042 to 0.676 g 100 g^−1^), but its content differed between species and cultivars. Meanwhile, this alkaloid was not found in leaf extracts, despite the fact that they exhibited higher antioxidant potential compared to twig extracts. Particularly high berberine content was found for two golden-leafed cultivars: ‘Golden carpet’ and ‘Golden Ring’ of the *B. thunbergii* species.

Preliminary in vitro studies showed that ornamental barberry twig extracts, compared to pure berberine sulfate administered in an analogous dose, show greater cytotoxicity towards studied HaCaT, A375, and Caco-2 cells. It was suggested that matrix effects related to the presence of other phytochemical components may be responsible for the observed greater bioactivity of berberine-rich extracts. These findings seem to be innovative due to the health effects of berberine being well documented, whereas the effects of ornamental barberry extracts with a more complex composition are less understood and pose a research challenge. However, due to the fact that the composition of the extract complex mixture is not fully established, conclusions about the bioactivity of the berberine-containing extract should be treated with caution. They need confirmation in the future planned comparative research of the cytotoxic effect against used cell lines between selected twig barberry extracts and pure berberine isolated from them. Valuable clarifications could also be obtained from future studies of the bioavailability of berberine from the barberry twig extract using in vitro digestion.

Due to the fact that the use of the full barberry extract without the need for berberine purification seems to be more economically advantageous, the next step of research will be the scaling up of the extraction technology for industrial applications. However, since ornamental barberry twigs are easier to obtain in controlled cultivation of this plant than common barberry roots, promising results of preliminary studies may lead to the offer of a new raw material for the isolation of berberine for the pharmaceutical industry as well as the use of the full extract of ornamental barberry twigs as a new form of dietary supplement. However, this requires deeper research on the mechanisms of berberine and berberine-containing extract action using various biological models.

## Figures and Tables

**Table 1:** Characterization of used *Berberis* spp. plants.

No.	Cultivar	Species	Leaf Color
1	‘Red Tears’	*Berberis koreana*	Green leaves, turning red in autumn
2	‘NN’	*Berberis koreana*	Green leaves, turn purple in autumn. Seedlings of unknown origin selected and cloned in ‘Więcek’ nursery
3	‘Superba’	*Berberis* × *ottawensis* (*B. thunbergii* × *B. vulgaris*)	Dark red leaves with a bluish tint
4	‘Powwow’	*Berberis thunbergii*	The leaves are yellowish at the beginning of the vegetation period, later becoming green, some with lighter spots. in autumn they turn orange-red
5	‘Golden Carpet’	*Berberis thunbergii*	Intense yellow leaves; in full sun the leaves may burn
6	‘Red Pillar’	*Berberis thunbergii*	Greenish-red leaves
7	‘Golden Ring’	*Berberis thunbergii*	Dark purple-red leaves with a greenish-yellow border

**Table 2:** Total phenolic content and antioxidant potential of analyzed plant material.

Cultivar	TPC [mg GAE g^−1^ d.m]		FRAP [μmol TE g^−1^ d.m.]		DPPH [μmol TE g^−1^ d.m.]	
	Leaves	Twigs	Leaves	Twigs	Leaves	Twigs
‘Red Tears’	63.91 ± 0.79 ^b^	19.19 ± 0.30 ^a^	293.47 ± 12.30 ^a^	83.69 ± 13.20 ^ab^	77.15 ± 11.88 ^a^	39.38 ± 0.46 ^a^
‘NN’	107.61 ± 1.30 ^c^	32.17 ± 2.33 ^c^	494.52 ± 13.05 ^bc^	152.87 ± 18.74 ^d^	328.00 ± 42.14 ^c^	98.02 ± 7.28 ^c^
‘Superba’	96.12 ± 5.87 ^c^	25.63 ± 1.24 ^b^	434.08 ± 9.97 ^b^	112.93 ± 12.20 ^c^	216.36 ± 44.63 ^b^	66.44 ± 6.48 ^b^
‘Powwow’	49.25 ± 2.70 ^a^	15.16 ± 1.09 ^a^	223.83 ± 12.85 ^a^	71.86 ± 1.90 ^a^	50.96 ± 4.88 ^a^	34.50 ± 0.38 ^a^
‘Golden Carpet’	104.24 ± 5.84 ^c^	24.25 ± 0.67 ^b^	500.22 ± 18.69 ^c^	116.35 ± 6.35 ^c^	314.58 ± 22.22 ^c^	56.77 ± 4.34 ^b^
‘Red Pillar’	103.35 ± 3.29 ^c^	19.76 ± 1.21 ^ab^	528.91 ± 5.38 ^c^	95.73 ± 6.35 ^bc^	216.11 ± 5.42 ^b^	43.76 ± 4.29 ^ab^
‘Golden Ring’	136.64 ± 6.12 ^d^	25.82 ± 1.49	686.82 ± 30.57 ^d^	113.09 ± 6.39 ^c^	301.17 ± 17.27 ^c^	67.87 ± 7.75 ^b^
*Berberis vulgaris* root bark	8.21 ± 0.23		35.62 ± 1.82		14.11 ± 0.17	
*Berberis vulgaris* (organic) root bark	5.81 ± 0.65		25.84 ± 0.77		9.50 ± 0.29	

^a,b,c,d^—means marked with different letters are significantly different (*p* < 0.05).

**Table 3:** Correlation matrix for the studied parameters of antioxidant activity, polyphenols, and berberine content.

	TPC	FRAP	DPPH	Berberine Content
TPC	1.000	0.982 *	0.978 *	−0.430
FRAP	0.982 *	1.000	0.970 *	−0.321
DPPH	0.978 *	0.970 *	1.000	−0.446
Berberine content	−0.430	−0.321	−0.446	1.000

*—marked correlation coefficients are significant (*p* < 0.05).

**Table 4:** The comparison of the cytotoxic effect of pure berberine and ornamental barberry twig extracts.

Tested Sample	Berberine Content [µg mL^−1^]	Viability [%]		
		HaCat	A375	Caco-2
Ornamental barberry extracts (500 µg mL^−1^ dose)				
‘Golden carpet’	16.3	63.59 ***	85.87 ***	87.55 **
‘Golden Ring’	11.83	72.89 ***	84.79 **	82.20 **
‘Superba’	3.9	85.24 ***	44.21 ***	93.66 *
‘Red Tears’	1.06	94.10	77.93***	76.42 ***
Pure berberine				
	1	96.80 ***	95.89 **	96.88
	10	82.80 ***	79.70 ***	95.89
	100	58.64 ***	40.32 ***	74.68 ***

Statistical significance according to control cells was assessed using one-way ANOVA and Dunnett’s post hoc test (* *p* < 0.05, ** *p* < 0.01, *** *p* < 0.001).

## Data Availability

Data are contained within the article.
